# The PDL1-inducible GTPase Arl4d controls T effector function by limiting IL-2 production

**DOI:** 10.1038/s41598-018-34522-4

**Published:** 2018-10-31

**Authors:** Felix Tolksdorf, Julita Mikulec, Bernd Geers, Jessica Endig, Paulina Sprezyna, Lukas C. Heukamp, Percy A. Knolle, Waldemar Kolanus, Linda Diehl

**Affiliations:** 1 LIMES (Life and Medical Sciences Institute), Molecular Immunology, University of Bonn, Bonn, Germany; 2 Institute of Molecular Medicine, University Hospital Bonn, Bonn, Germany; 3 Division of Virus-Associated Carcinogenesis (F170), German Cancer Research Center (DKFZ), Heidelberg, Germany; 4 Institute of Experimental Immunology and Hepatology, University Medical Center Hamburg-Eppendorf, Hamburg, Germany; 5 Institute of Pathology, University of Cologne, Cologne, Germany; 6 Institute of Molecular Immunology and Experimental Oncology, Technical University Munich, Munich, Germany

## Abstract

Interleukin-2 (IL-2) is a key regulator of adaptive immune responses but its regulation is incompletely understood. We previously found that PDL1-dependent signals were pivotal for liver sinusoidal endothelial cell-mediated priming of CD8 T cells, which have a strongly reduced capacity to produce IL-2. Here, we show that the expression of the ARF-like GTPase Arl4d is PD-L1-dependently induced in such LSEC-primed T cells, and is associated with reduced IL-2 secretion and Akt phosphorylation. Conversely, Arl4d-deficient T cells overproduced IL-2 upon stimulation. Arl4d-deficiency in CD8 T cells also enhanced their expansion and effector function during viral infection *in vivo*. Consistent with their increased IL-2 production, Arl4d-deficient T cells showed enhanced development into KLRG1^+^CD127^−^ short-lived effector cells (SLEC), which is dependent on IL-2 availability. Thus, our data reveal a PD-L1-dependent regulatory circuitry that involves the induction of Arl4d for limiting IL-2 production in T cells.

## Introduction

In recent years it has become increasingly clear that the T cell growth factor interleukin-2 (IL-2) exerts multiple functions in regulation of the immune system^1,2^. IL-2, for instance, is pivotal for both the development of CD4^+^CD25^+^ regulatory T cells (T_reg_)^3,4^ and their immune suppressive function. Mature T_reg_ limit T cell responses directly via the consumption of IL-2^5,6^ or indirectly by limiting IL-2 production due to inhibited DC maturation^7^. Also, IL-2 dependent signals promote T_H_1, T_H_2 and T_reg_ development but counteract T_H_17 and T_FH_ differentiation^8^. Furthermore, IL-2 is crucial for both the induction of primary CD8 T cell responses^9,10^ as well as the optimal induction and secondary expansion of memory CD8 T cells^11,12^. For instance, the amount of IL-2 produced by CD8 T cells after priming affects their capacity for clonal expansion^13,14^. Specifically, the level and duration of naïve CD8 T cell exposure to IL-2 influences their development into short-lived effector cells, responsible for acute pathogen clearance, or long-lived memory T cells^15,16^. T cells lacking the IL-2Rα form fewer KLRG1^+^ SLEC during viral infection^15^. Conversely, during viral infection a subset of CD8 T cells expressing high levels of CD25 preferentially develop into terminally differentiated effector cells^16^.

Not only is IL-2 availability regulated by co-stimulation or consumption by T_reg_, its production is actively limited via co-inhibitory programmed-death-1 (PD-1) in T cells^17,18^. Signalling via PD-1 can prevent Akt phosphorylation via inhibition of PI3K activity^19^, which in CD8 T cells precludes the generation of effector phenotype and function^20,21^. Similarly, during priming of naïve CD8 T cells by liver sinusoidal endothelial cells (LSEC), LSEC-derived PD-L1 signals^22,23^ and the resulting limited production of IL-2^24^ are pivotal for the development of liver-primed T cells, which although unable to directly mediate effector function^23,25^, are capable of memory function providing anti-infectious immunity^26^.

Here, we describe a new molecule regulated by PD-1 signalling in CD8 T cells. The ADP ribosylation factor (ARF)-like GTPase Arl4d is upregulated in liver-primed T cells, which are strongly defective in IL-2 production. Significantly, priming of T cells by LSEC devoid of PD-L1 expression restores specific antigen-induced IL-2 production while at the same time failing to induce high expression of Arl4d. Reversely, Arl4d-deficient CD8 T cells show enhanced IL-2 production and maturation into effector cells during viral infection *in vivo*. Thus, our data establish the ARF-like GTPase Arl4d as a novel component of a regulatory pathway induced by PD-L1, which regulates IL-2 production and controls adaptive CD8 T cell responses in the immune system.

## Results

### Expression of Arl4d in CD8 T cells is induced by LSEC, but not DC, in a PD-L1-dependent fashion

**Figure 1:**
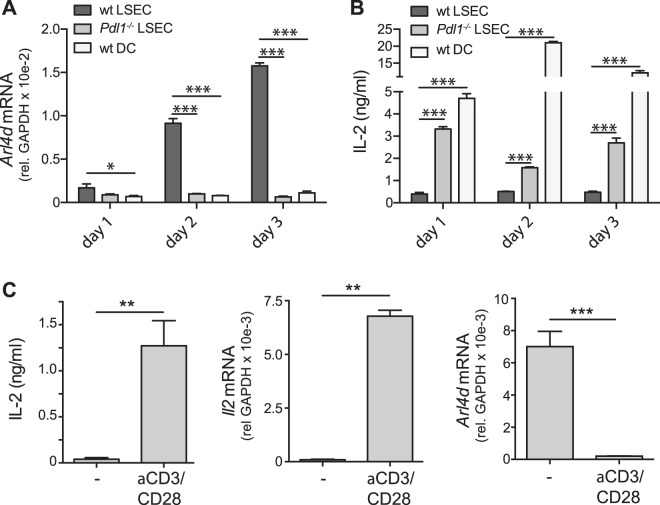
Arl4d expression is PD-L1/PD-1 dependently regulated in CD8 T cells. (**A**,**B**) Naive OT-1 CD8 T cells were cultured for the indicated times on C57BL/6 (wild type) LSEC, *Pdl1*^−*/*−^ LSEC or C57BL/6 (wild type) DC in the presence of antigen (100 μg/ml OVA). (**A**) relative *Arl4d* mRNA expression levels in CD8 T cells. (**B**) IL-2 concentration in the culture supernatant. (**C**) Wild type CD8 T cells were cultured in the presence or absence of coated anti-CD3ε/CD28 antibodies. After 24 h T cells were harvested and *Arl4d* and *Il2* mRNA levels were determined by qPCR and IL-2 content in the supernatant by ELISA. The data shown are representative of 3 separate experiments. Data are shown as mean +/− s.e.m. Statistical significance was calculated using a one-way ANOVA, * p ≤ 0.05, ** p ≤ 0.01, ***p ≤ 0.001.

We have previously shown that during LSEC-mediated priming of CD8 T cells, which leads to the induction of CD8 T cells incapable of immediate effector function^22,26^, insufficient IL-2 production during priming is essential^24^. Gene expression analysis of *in vitro* LSEC- and DC-primed CD8 T cells revealed expression of Arl4d, but not its family members Arl4a or Arl4c, to be induced in LSEC-primed, but not DC-primed CD8 T cells (data not shown). Quantitative real-time PCR of T cells primed by LSEC, *Pdl1*^−*/*−^ LSEC and dendritic cells (DC) confirmed that *Arl4d* mRNA was potently induced during LSEC-mediated CD8 T cell stimulation (Fig. 1A). However, in the absence of PD-L1-dependent signals or during priming by DC, *Arl4d* mRNA levels in CD8 T cells were not. The increased levels of *Arl4d* mRNA in LSEC-primed CD8 T cells correlated with a decreased IL-2 production, whereas *Pdl1*^−*/*−^ LSEC and DC-stimulated T cells with low *Arl4d* mRNA content produced high levels of IL-2 (Fig. 1B). Although both in DC- and *Pdl1*^−*/*−^ LSEC-stimulated T cells *Arl4d* mRNA levels are equally low, DC induce more IL-2 secretion by in T cells than *Pdl1*^−*/*−^ LSEC. Compared to LSEC^22^, however, DC express high levels of CD80 and CD86, most likely leading to the observed increased IL-2 production. Furthermore, in polyclonal wild type CD8 T cells stimulated with anti-CD3 and anti-CD28 antibodies, *Arl4d* mRNA levels were markedly reduced after activation, which again correlated with T cells gaining the ability to produce IL-2 (Fig. 1C). Thus, these data indicate that PD-L1/PD-1, which is pivotal for preventing the development of effector function in T cells stimulated by LSEC, augments Arl4d expression in T cells.

### Arl4d negatively regulates Akt phosphorylation in activated T cells

**Figure 2:**
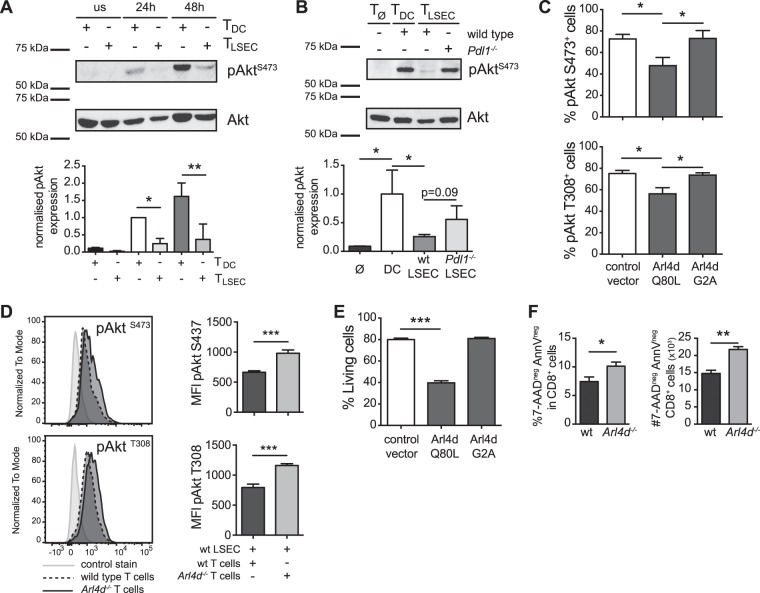
Arl4d interferes with Akt phosphorylation in activated T cells. (**A**,**B**,**D**,**F**) naïve OT-1 CD8 T cells from wild type (**A**,**B**,**D**,**F**) and *Arl4d*^−*/*−^ OT-1 T cells (**D**,**F**) were co-cultured with antigen-loaded wild type DC, wild type LSEC or *Pdl1*^−*/*−^ LSEC as indicated. (**A**,**B**) At the indicated times T cells were analysed by western blot for expression of pAkt^S473^ and total Akt. Shown are cropped images of the blot (full length blots are presented in Supplementary Figure S2). Bar graphs show normalised expression levels of pAkt. (**C**,**E**) A constitutive active Arl4d mutant (Q80L), a myristoylation-deficient Arl4d mutant (G2A) or an empty pEGFP vector were expressed in Jurkat T cells. (**C**) Jurkat cells were stimulated with anti-CD3 antibodies and analysed for expression of phosphorylated Akt (S473, T308) by flow cytometry in GFP^+^ Jurkat T cells (n = 5). (**D**) wt and *Arl4d*^−*/*−^ T cells stimulated by wild type LSEC for 48 h were stained for pAkt^S473^ and pAkt^T308^ Representative histograms and mean fluorescence intensity (MFI) of CD8^+^ cells are shown. (**E**,**F**) Survival of Jurkat cells (**E**) 24 h after transfection with Arl4d-mutant constructs or (**F**) wild type and *Arl4d*^−*/*−^ OT-1 T cells stimulated by wild type LSEC. Cells were stained with Propidium iodide or 7-AAD and Annexin V. Live cells were defined as being GFP^pos^/CD8α^pos^ and AnnV^neg^ and PI/7-AAD^neg^. The data shown are representative of 3 (**A**,**B**), 5 (**C**,**D**,**E**) and 2 (**F**) separate experiments. Data are shown as mean +/− s.e.m. Statistical significance was calculated using a one-way ANOVA or a Student’s *t*-test, *p ≤ 0.05, **p ≤ 0.01, ***p ≤ 0.001.

During T cell activation, TCR triggering together with CD28 co-stimulation activates the PI3K/Akt pathway leading to complete T cell activation and initiation of IL-2 production^27^. PD-1 can repress this process due to downstream inhibition of the PI3K/Akt pathway^19^. Indeed, when we compared Akt phosphorylation in T cells primed by dendritic cells with T cells primed by LSEC, we observed an increasing amount of Akt phosphorylation in DC-primed T cells (Fig. 2A). In contrast, in LSEC-primed CD8 T cells phosphorylated Akt was almost absent (Fig. 2A). The low levels of pAkt in LSEC-primed T cells was mediated via PD-L1 signals as in T cells primed by *Pdl1*^−*/*−^ LSEC high levels of pAkt could be detected (Fig. 2B), revealing an inverse correlation of Arl4d expression with the amount of pAkt in T cells. Moreover, Arl4d directly modulated Akt phosphorylation. Overexpression in Jurkat T cells of a constitutively active Arl4d^Q80L^ reduced pAkt content, whereas the myristoylation-deficient Arl4d^G2A^ did not (Fig. 2C). These data indicate that binding to GTP and localisation to membranes by Arl4d are important for interference with Akt function. We confirmed that the constructs used were expressed equally (Suppl. Figure S1), excluding transfection efficiency differences for the observed results. Reversely, in Arl4d-deficient T cells stimulation by wild type LSEC resulted in higher pAkt S437 and T308 levels as in wild type T cells (Fig. 2D), indicating that in wild type T cells the induction of Arl4d via PDL1-signalling from LSEC leads to reduction of Akt signalling.

As Akt is an important regulator of cellular viability by regulating survival and apoptotic pathways^28,29^, we analysed whether cell viability was changed upon overexpression of Arl4d. In Jurkat T cells, compared to the eGFP vector control, overexpression of Arl4d^Q80L^ strongly reduced the number of living (AnnV^neg^/PI^neg^) T cells (Fig. 2E), whereas Arl4d^G2A^ did not, indicating that Arl4d may regulate T cell survival via modulation of Akt phosphorylation status. Again, the opposite could be observed in *Arl4d*^−*/*−^ T cells (Fig. 2F), where the absence of Arl4d led to increased survival of T cells upon stimulation by LSEC. Thus, Arl4d plays a role in regulating Akt activity and survival in T cells via a membrane-proximal, GEF-dependent mechanism.

### Arl4d-deficiency does not affect T cell development but leads to increased IL-2 secretion

**Figure 3:**
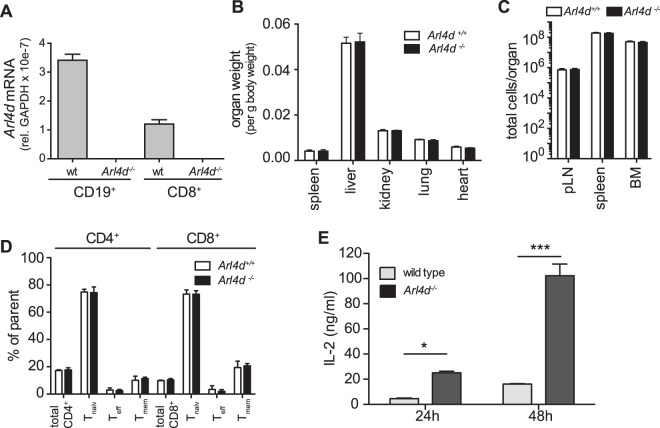
Arl4d-deficiency does not affect T cell development, but leads to enhanced IL-2 production. (**A**) CD8^+^ T cells and CD19^+^ B cells were isolated from the spleen of *Arl4d*^−*/*−^ mice and their wild type littermate controls. qPCR was performed to determine *Arl4d* mRNA expression. (**B**) Organ weights of *Arl4d*^−*/*−^ mice (n = 6) and their wild type littermate controls (n = 6). (**C**) Total cell counts in different lymphatic (peripheral lymph node, spleen) and the bone marrow in *Arl4d*^−*/*−^ mice (n = 6) and their wild type littermate controls (n = 6). (**D**) Analysis of the T cell compartment in the spleen from 6–8 week old *Arl4d*^−*/*−^ mice (n = 6) and their wild type littermate controls (n = 6). Cells were gated according to CD4 or CD8 expression and according to their expression of CD44 and CD62L (T_naiv_: CD44^low^CD62L^high^, T_eff_: CD44^high^CD62L^low^, T_mem_: CD44^high^CD62L^high^). (**E**) *Arl4d*^−*/*−^ and wild type CD8 T cells were stimulated with plate-bound anti-CD3ε (1 μg/ml) and anti-CD28 (10 μg/ml) antibodies for the indicated times, after which IL-2 content in the supernatant was assed by ELISA. The data shown are representative of 3 (**A**) and 4 (**E**) separate experiments, respectively, and shown as mean +/− s.e.m. Statistical significance was calculated using a two-way ANOVA, *p ≤ 0.05, **p ≤ 0.01, ***p ≤ 0.001.

In order to investigate the role of Arl4d in T lymphocytes, we made use of Arl4d-deficient mice (Arl4d^tm1a(EUCOMM)Wtsi^), which were generated by a knock-out-first approach^30^. We confirmed that Arl4d expression was indeed absent in these mice by analysing *Arl4d* mRNA levels in CD19^+^ and CD8^+^ cells from *Arl4d*^−*/*−^ and *Arl4d*^+/+^ animals (Fig. 3A) We then analysed their general development and immune system with a focus on T cell development. Arl4d-deficiency neither affected organ weight (Fig. 3B), nor the cellularity of various lymphoid organs or the bone marrow (Fig. 3C). Additionally, a detailed analysis of the T cell compartment, by staining for CD4, CD8, CD62L and CD44, showed that T cell development and their distribution into naïve (CD62L^high^CD44^low^), effector (CD62L^low^CD44^high^) and memory (CD62L^high^CD44^high^) subsets in the periphery was not changed in the absence of Arl4d expression (Fig. 3D). Thus, general development of *Arl4d*^−*/*−^ mice and their T cell compartment is not changed. In contrast, when Arl4d-deficient T cells were activated via CD3 and CD28, IL-2 secretion was significantly increased compared to the wild type control T cells (Fig. 3E), indicating that although Arl4d is not involved in T cell development, it represses IL-2 production upon T cell activation.

### Arl4d-deficiency leads to an enhanced CD8 T cell response and CD8-mediated IL-2 production after viral infection *in vivo*

**Figure 4:**
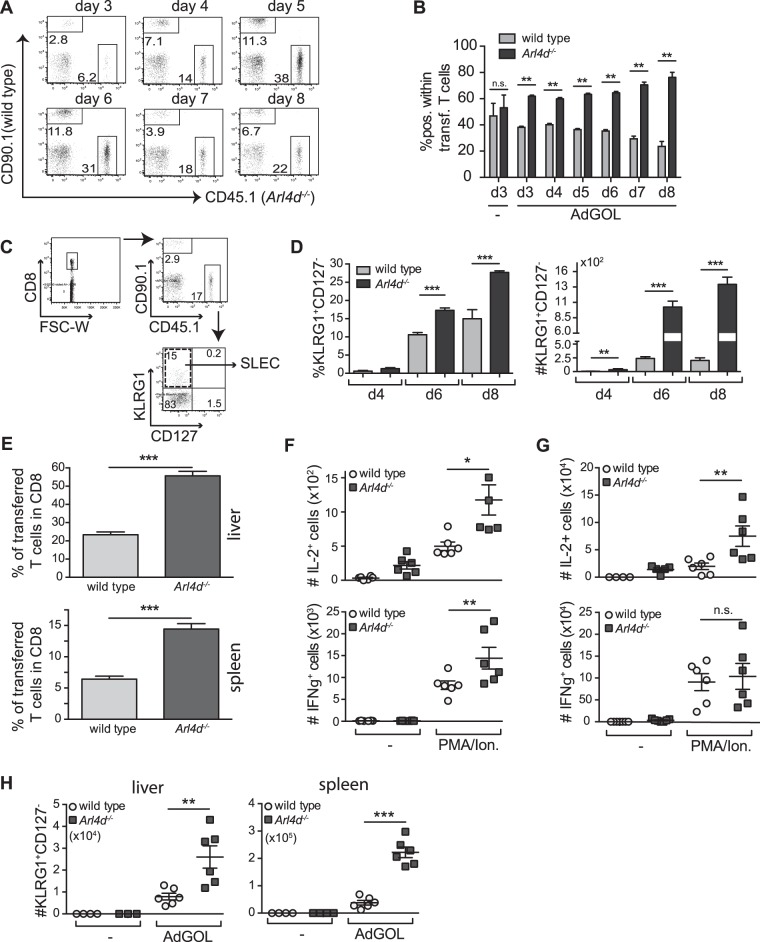
Enhanced expansion and effector cell differentiation of Arl4d-deficient CD8 T cells upon viral infection. Wild type CD90.1 OT-1 CD8 T cells and *Arl4d*^−*/*−^ CD45.1 OT-1 T cells were adoptively transferred into CD45.2 congenic recipient mice in a 1:1 ratio, which 1 day later were infected with an adenovirus expressing OVA (AdGOL) (n = 6) or were left untreated (n = 4). (**A**) Expansion of adoptively transferred T cells. Dot plots are gated on total CD8 T cells. (**B**) Relative contribution of wild type and *Arl4d*^−*/*−^ CD8 T cells to the total population of transferred CD8 OT-1 T cells during AdGOL infection in blood. (**C**) Gating strategy for short-lived effector cells (SLEC): CD8^+^CD127^-^KLRG1^+^. (**D**) Percentages and absolute numbers of CD8^+^CD127^-^KLRG1^+^ SLEC in the blood within the transferred wild type and *Arl4d*^−*/*−^ OT-1 T cells at the indicated times after AdGOL infection. (**E**) 8 days after AdGOL infection lymphocytes were isolated from the liver and spleen and the percentage of transferred wild type OT-1 and *Arl4d*^−*/*−^ OT-1 T cells was determined within the total CD8^+^ T cell population. (**F**,**G**) Absolute numbers of cytokine producing CD8 T cells 8 days after infection with AdGOL within the transferred T cell population from liver (**F**) and spleen (**G**) 4 h after PMA/ionomycin restimulation. (**H**) Absolute numbers of CD8^+^CD127^-^KLRG1^+^ SLEC in the spleen and liver 8 days after AdGOL infection within the transferred T cell population. The data shown are representative of 3 separate experiments. Data are shown as mean +/− s.e.m. Statistical significance was calculated using a one-way ANOVA, *p ≤ 0.05, **p ≤ 0.01, ***p ≤ 0.001.

IL-2 is a major regulator of immune responses *in vivo*^8^. To determine if the inhibitory effects of Arl4d on IL-2 production in T cells would have an effect on *in vivo* immunity, we co-transferred equal amounts of sorted CD8^+^CD62L^high^CCD44^low^ Arl4d-deficient (CD45.1) and wild type (CD90.1) naïve OT-1 CD8 T cells into congenic recipients and followed their expansion and function upon infection with an OVA-expressing adenovirus (AdGOL). From day 3–4 onwards the adoptively transferred CD8 T cells could be detected in the blood of congenic wild type recipients infected with AdGOL (Fig. 4A). Interestingly, the *Arl4d*^−*/*−^ CD8 T cells expanded to a larger extent than their wild type counterparts leading to an almost 80% contribution of *Arl4d*^−*/*−^ CD8 T cell to the anti-viral immune response within total transferred T cells in the circulating blood (Fig. 4B). It is further known that in response to viral or bacterial infection antigen-specific CD8 T cells differentiate into effector cells that can be subdivided into short lived effector cells (SLEC), which are terminally differentiated effector cells, and memory precursor effector cells (MPEC) that develop into memory T cells. The development of SLEC can be promoted by the pro-inflammatory mediators IL-12^31^ and Type I IFN^32^ via the induction of T-bet expression. Additionally, also the availability of IL-2^33^ or the capability of T cells to respond to IL-2^12,34^ contributes to the fate-decision of effector T cells to become SLEC. Within the *Arl4d*^−*/*−^ CD8 T cell population significantly more CD8^+^CD127^-^KLRG1^+^ SLEC accumulated during adenoviral infection compared to the wild type T cell population (Fig. 4D). Not only did *Arl4d*^−*/*−^ T cells contribute to a larger extent to the circulating T cell pool during adenovirus infection, also 8 days after adenoviral infection, the Arl4d-deficient T cells expanded significantly more than their co-transferred wild type counterparts within the spleen and the primary infection site, i.e. the liver (Fig. 4E). Functionally, Arl4d-deficiency led to enhanced numbers of IL-2 producing effector T cells in the liver (Fig. 4F) and spleen (Fig. 4G), respectively. This effect appears to be specific for IL-2 as Arl4d-deficient IFNγ-producing cells were also increased in the liver but not in the spleen. Moreover, also in the spleen and the infection site liver, development of *Arl4d*^−*/*−^ T cells into KLRG1^+^CD127^−^ SLEC was more pronounced (Fig. 4H), similar to the observation in the blood. Thus, overall Arl4d has a dampening effect on the CD8 T cell-mediated anti-viral immune response as its deletion in T cells leads to greater expansion of effector cells, higher SLEC development and enhanced cytokine production upon secondary stimulation.

## Discussion

In this study we introduce the Arf-like GTPase Arl4d as a novel factor in PD-L1-dependent regulation of lymphocyte function. We find that Arl4d is expressed in naive CD8 T cells and is regulated during T cell activation. In particular, TCR triggering in the presence of costimulation, such as priming by dendritic cells or also with CD3/CD28-antibodies does not affect or even reduces Arl4d expression whereas TCR triggering in combination with coinhibition (PD-L1), in the case of priming by liver sinusoidal endothelial cells, results in its marked upregulation. This PD-L1-dependent induction of Arl4d during T cell priming by LSEC and its effects on IL-2 production suggests that this protein complex is involved in PD-1-mediated attenuation of T cell function. Central to PD-L1/PD-1-mediated inhibition of T cell function is the inhibition of the PI3K/Akt pathway^35^ via binding of SHP2 to the intracellular ITSM motif in PD-1^19,36^. Arl4d appears to directly influence this pathway, as its overexpression led to reduced pAkt in Jurkat T cells and additionally led to increased levels of pAkt in LSEC-primed Arl4d-deficient T cells.

The inhibition of IL-2 production associated with Arl4d upregulation may indirectly cause insufficient Akt phosphorylation induced via the IL-2 receptor CD25, leading to an inability to induce transcriptional programs for cytotoxic activity in CD8 T cells^20^. Indeed, the lack of Arl4d results in higher IL-2 production and more pronounced effector cell development, as measured by an increase in KLRG1^pos^CD127^neg^ short-lived effector CD8 T cells during viral infection *in vivo*. This is in line with reports showing enhanced expansion of CD8 T cells in the presence of higher levels of IL-2^10,14^ and increased development of SLEC due to IL-2-induced Blimp-1 expression^37,38^. Although Arl4d-deficient T cells do produce more IL-2 and display higher expansion and SLEC development, the direct effect of Arl4d expression in down-modulating the PI3K-Akt signalling pathway may also play a role. In adenovirus-infected mice, both Arl4d-deficient and -proficient T cells shared the same space, and thus each cell type may have benefitted from factors produced by the other. Although autocrine effects are described to be more important for IL-2 function, it has also been reported that paracrine IL-2 production can have direct effects on CD8 T cell survival and proliferation^13^. Thus, Arl4d-proficient T cells may have profited from enhanced IL-2 levels *in vivo* due to the overproduction by *Arl4d*^−*/*−^ T cells as well. Here, Arl4d-deficiency was still advantageous in terms of expansion and effector function (IL-2, IFNγ production), suggesting that not only increased IL-2 but also intrinsic Arl4d-mediated inhibition of intracellular signalling may play a role in repression of T cell function.

Arl4d is a member of the family of ARF-like GTPases, which also includes Arl4a and Arl4c. So far, not much is known about its function. Some reports show involvement of Arl4d in adipogenesis^39^, actin remodelling^40^ and neurite outgrowth^41^. Structurally, it contains a nuclear localisation site (NLS) and a myristoylation site, mediating nuclear and membrane localisation, respectively^42^. Membrane targeting of Arl4d via myristoylation is a necessary step in order to allow for a conformational change that permits GTP binding. Forcing Arl4d to stay in a GDP-bound form via mutagenesis leads to a different localisation, i.e. to the mitochondrial membrane, where Arl4d-GDP can interfere with mitochondrial membrane potential^43^. However, for its inhibitory function in T cells we found that membrane targeting via myristoylation is necessary. A mutant Arl4d, that cannot be myristoylated (Arl4d^G2A^), was not able to interfere with Akt phosphorylation when overexpressed in Jurkat T cells.

Arl4d and its family members have further been shown to be involved in the membrane recruitment of members of the cytohesin family^42^. In particular, the recruitment of cytohesin-2, also called ARNO, by Arl4d leads to further downstream effects on actin dynamics, via recruitment of another Arf GTPase Arf6^40^. Whether Arl4d expression can modulated actin dynamics in T cells remains to be investigated, but it could have profound effects on T cell function. Actin dynamics are reported to be involved in T cell migration, but also TCR signal transduction itself could be influenced, as the actin cytoskeleton promotes the formation of the immune synapse and the transport of various molecules (including the TCR) into the synapse, which is the hub for signal transduction initiation and termination^44,45^.

Our data point to a role for Arl4d in T cell activation and effector cell differentiation. More specifically, our data shown that Arl4d interferes with signal transduction via the PI3K/Akt axis, leading to inhibition of cytokine production (IL-2) and suppression of SLEC development during viral infection. Arl4d expression appears to be regulated via co-signalling that is integrated after TCR stimulation. Co-stimulatory signalling via CD28 reduces Arl4d expression, whereas co-inhibitory PD-1 signalling markedly up regulates Arl4d expression in T cells. Thus, our data identify Arl4d as a new factor involved in PD-1 mediated signalling. The exact molecular mechanisms by which Arl4d confers inhibitory signalling in T cells remain to be investigated, but may reveal additional insight into PD-1-dependent co-inhibitory signalling.

## Methods

### Mice

Arl4d^tm1a(EUCOMM)Wtsi^,^30^, CD45.1, CD90.1, *B7H1*^−*/*−^ (*Pdl1*^−*/*−^)^46^, C57BL/6 J and OT-1 (C57BL/6-^Tg(TcraTcrb)1100Mjb^/J) mice were bred and backcrossed in the animal facilities of the University Hospital Bonn, the LIMES institute and the University Medical Center Hamburg-Eppendorf according to the Federation of European Laboratory Animal Science Association guidelines and maintained under SPF conditions. All mouse experiments were approved by the local authorities of Nordrhein-Westfalen and Hamburg (84-02.04.2013.A237, G30/15) and carried out according to the current existing guidelines on mouse experimentation.

### Cell isolation

Liver sinusoidal endothelial cells were isolated as previously described^22^. Dendritic cells were isolated from collagenase-digested spleens using CD11c Microbeads (Miltenyi Biotech). Naïve CD8 T cells were isolated using the CD8 T cell Isolation kit (Miltenyi Biotech, Bergisch Gladbach, Germany) according to the manufacturer’s recommendations.

### Quantitative real-time PCR

RNA was isolated from cells with an RNeasy plus kit (Qiagen) and cDNA synthesis was performed with a Superscript VILO kit (Invitrogen) or a High-Capacity cDNA reverse transcription kit (Applied Biosystems). qRT-PCR was performed using specific primers and a Maxima SybrGreen/Rox master mix (Thermo Scientific). Primers used were muIL-2: fw 5′AACCTGAAACTCCCCAGGAT-3′, rv 5′-TCATCATCGAATTGGCACTC-3′, muArl4d: fw 5′GTCGTCATTGGGTTGGATTC-3′, rv 5′-ACTTGGAAAGTGATCCCACG-3′, muGapdh: fw 5′-GAGAAACCTGCCAAGTATGATG-3′, rv 5′-GTCATACCAGGAAATGAGCTTG-3′.

### Western blot

Cells were pelleted and washed with PBS before lysis in lysisbuffer (50 mM Tris-HCL, 1 mM EGTA, 1 mM EDTA, 10 mM β-Glycerol-phosphate, 50 mM NaFl, 5 mM pyrophosphate, 1 mM Na-orthovanadate, 0,270 M sucrose, 1% triton). Protein content was assessed by the BCA method and equal amount of protein were loaded onto SDS page gels for separation. Proteins were blotted onto nitrocellulose membranes and stained with anti-Akt S473 and total Akt, (Cell Signaling Technologies), washed, stained with an HRP-coupled secondary antibody and developed using the ECL method (Pierce).

Band intensities were measured with Image studio light 5.2 (Licor) and the strongest was set to 1 and all others in relation to this. Then a ratio was calculated between pAkt and total Akt from the same sample ( = normalised pAkt expression).

### Cloning, Overexpression

Wild type Arl4d was cloned from RNA derived from LSEC-primed CD8 T cells. Mutants were generated via introduction of mutations via PCR, which were validated via sequencing. Arl4d-G2A lacks the G at position 2 necessary for myristoylation and membrane targeting and the Arl4d-Q80L is a constitutive active form, which is unable to catalyse GTP to GDP. These constructs were subcloned into pEGFP vectors (Clontech) for transfection into Jurkat T cells. To this end Jurkat T cells were incubated with 20 μg plasmid DNA, transferred into a 4 mm cuvette and electroporated (exponential, 240 V, 1500 μF) using a Gene Pulser X Cell (Biorad). 24 h to 48 h later cells were used in experiments.

### *In vivo* adenoviral infection

4 × 10^5^ sorted naive CD8^+^, CD62L^high^, CD44^low^ T cells from spleens of OT-1 × *Arl4d*^−*/*−^ mice and wild type CD90.1 OT-1 were adoptively transferred in a 1:1 ratio i.v. into CD45.1 recipient mice. One day later mice were infected i.v. with 5 × 10^6^ pfu AdGOL, a recombinant adenovirus expressing GFP, OVA and Luciferase^47^. After infection 32 μl of blood was taken from the tail-vein at the indicated times and analysed by flow cytometry. Eight days after infection mice were sacrificed and liver lymphocytes and splenocytes were isolated for *in vitro* analysis. Cells were stained with antibodies against CD45.2, CD45.1, CD90.1, CD8, CD44, CD62L, KLRG1, CD127 and a live/dead stain (Hoechst 33258 (Sigma), near-IR dead cell stain kit or LIVE/DEAD fixable aqua dead stain (Thermo Fischer Scientific)). Fc-block (clone 2.4G2) was added in each staining. To enumerate cells a fixed amount of counting beads was added to the samples prior to acquisition.

### Assessment of T cell function

Splenocytes or liver lymphocytes isolated from AdGOL infected mice were restimulated using PMA (5 ng/ml; Sigma Aldrich) and Ionomycin (200 ng/ml, Sigma Aldrich) for 4 h in the presence of Brefeldin A and Monensin (eBioscience) after which they were analysed for cytokine production by intracellular staining. To assess cytokine production upon activation of naïve CD8 T cells, *Arl4d*^−*/*−^ or wild type CD8^+^ T cells were isolated from the spleen and stimulated in anti-CD3ε/CD28 coated plates (1 μg/ml and 10 μg/ml respectively) or PMA/ionomycin.

### Flow cytometry

Flow cytometric analyses were conducted on a Canto II or LSR II (BD Biosciences) and data were analysed using FlowJo software (Tree Star, Ashland, OR). Hoechst 33258 (Sigma) or LIVE/DEAD Fixable Violet or Near-IR Dead Cell Stain kit (Invitrogen) was used to exclude dead cells in all samples analysed. Anti-CD16/32 antibody (2.4G2) was included in each staining at 10 μg/ml to block unspecific antibody binding via Fc receptors. All antibodies were purchased from Biolegend or eBioscience. For intracellular cytokine staining, cells were fixed in 4% PFA and intracellular staining with fluorochrome-labelled antibodies was performed in Permeabilisation Buffer (eBioscience) according to the manufacturer’s protocol. Quantification of T cell numbers was performed using fluorochrome-labeled microbeads (CountBright absolute counting beads, Life Technologies). For intracellular pAkt staining cells were stained with surface markers, fixed in 4% paraformaldehyde, permeabilised in 90% ice-cold methanol and stained with pAkt^S473^ (#4060) and pAkt^T308^ (#13038) and a secondary anti-rabbit Alexa647 coupled antibody (#4414) (Cell Signalling Technologies). For viability assays, cells were stained with propidium iodide or 7-AAD together with Annexin V. Viable cells were defined as PI/7-AAD negative and AnnV negative. Data were analysed using Flow Jo software (Tree Star Inc.; CA, USA).

### Statistical analyses

Statistical analyses were performed with GraphPad Prism 6.0 (GraphPad Software inc.). Student’s *t*-test or ANOVA was used as indicated in the legends. Data are shown as mean +/− standard error of the mean, and statistical significance is indicated by *p ≤ 0.05, **p ≤ 0.01, ***p ≤ 0.001.

## Electronic supplementary material

Supplementary materials

## Data Availability

Data, materials and detailed protocols will be made available upon request.
