# Supplementary material for: The PDL1-inducible GTPase Arl4d controls T effector function by limiting IL-2 production
Source: Sci Rep. 2018 Oct 31;8:16123. doi: 10.1038/s41598-018-34522-4 (PMC6208435; doi:10.1038/s41598-018-34522-4)
Supplement: Supplementary file 1 — Supplementary materials [file 41598_2018_34522_MOESM1_ESM.pdf]

Supplementary materials for

The PDL1-inducible GTPase Arl4d controls T effector function by limiting IL-2 production.

Felix Tolksdorf, Julita Mikulec, Bernd Geers, Jessica Endig, Paulina Sprezyna,
Lukas C. Heukamp, Percy A. Knolle, Waldemar Kolanus and Linda Diehl

Supplementary Figure 1

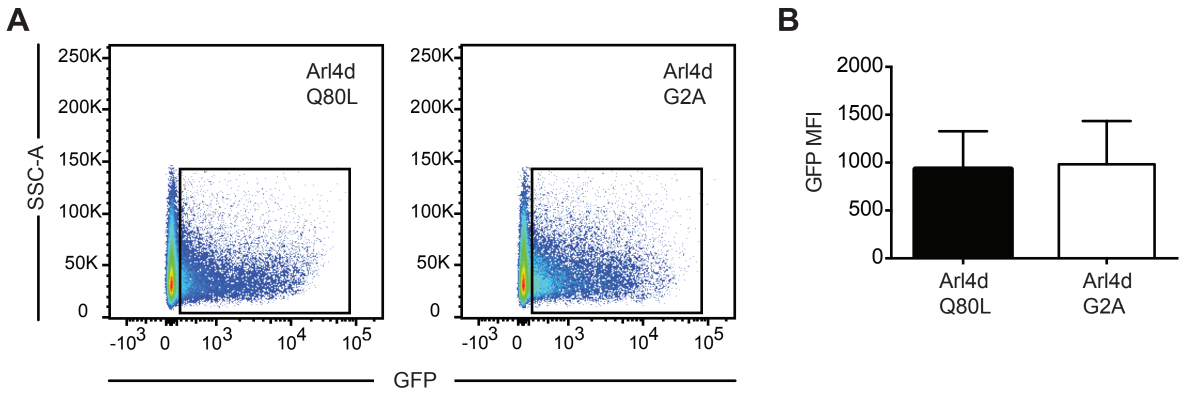

Supplementary Figure 1. Transfection efficiency of Arl4d constructs in Jurkat T cells. A: Exemplary dot plot of Jurkat T cells transfected with the Arl4d^{Q80L} and Arl4d^{G2A} mutants coupled to GFP. B: Average mean fluorescence index of GFP expression (n=5) in Arl4d^{Q80L} and Arl4d^{G2A} transfected Jurkat T cells. Data are shown as mean +/- s.e.m. Statistical significance was calculated using a Student's *t* test, * $p \leq 0.05$, ** $p \leq 0.01$, ***$p \leq 0.001$.

Supplementary Figure 2

x = 75kD

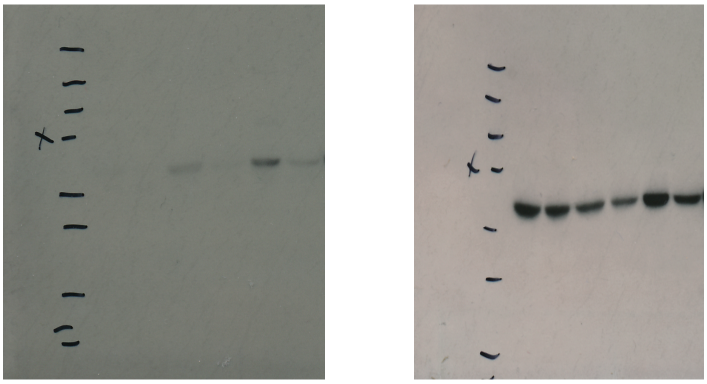

Original pAkt Blot
of Figure 2A

Original total Akt Blot
of Figure 2A

x = 75kD

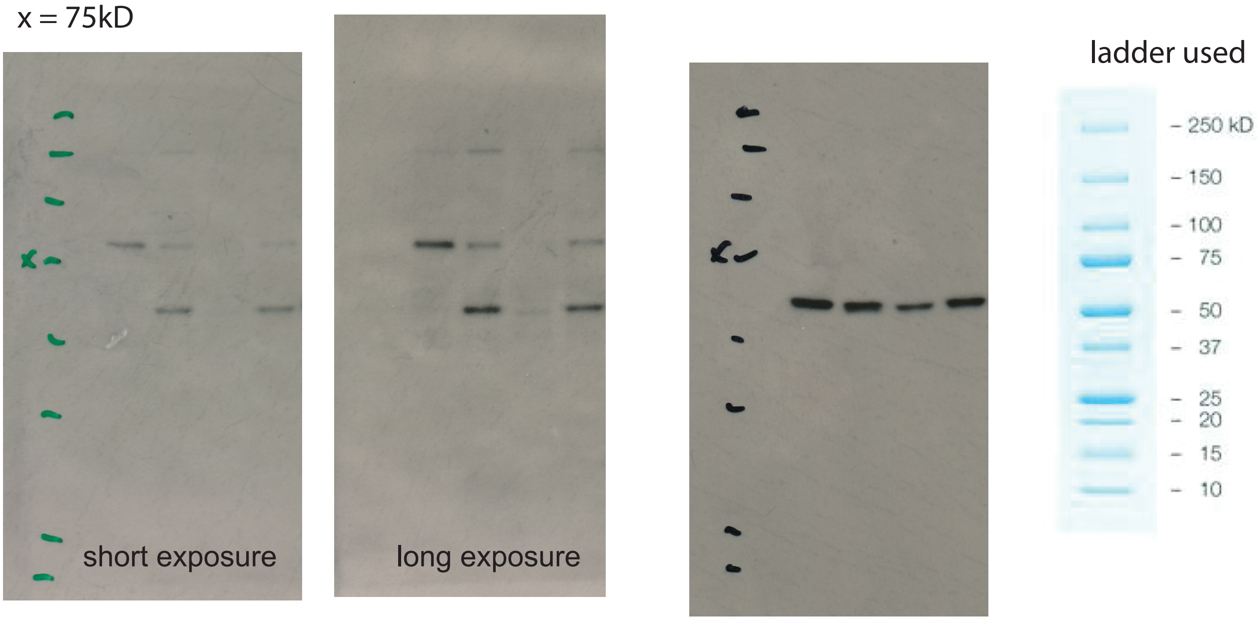

Original pAkt Blot of Figure 2B

Original total Akt Blot
of Figure 2B

Supplementary Figure 2. Original blots detecting pAkt and total Akt from Figure 2A and B. The ladder, which can only be seen on the membrane, was copied onto the ECL-developed blots with a marker pen and a cross on the blot next to a line indicates the 75kD band of the ladder.
